# Cross-Linking the TCR Complex Induces Apoptosis in CD4^+^8^+^ Thymocytes in the Presence of Cyclosporin A

**DOI:** 10.1155/1996/57362

**Published:** 1996

**Authors:** Heather L. Poetschke, David B. Klug, Dawn Walker, Ellen R. Richie

**Affiliations:** The University of Texas M.D. Anderson Cancer Center Science Park-Research Division P.O. Box 389 Smithville Texas 78957 USA

**Keywords:** Apoptosis, cyclosporin A, negative selection, thymocytes

## Abstract

Although it is generally agreed that TCR ligation is a minimal requirement for negative selection in the CD^+^8^+^ double-positive (DP) thymocyte subset, the costimulatory requirements and specific signaling events necessary to induce apoptosis are not well defined. We have explored the consequences of cross-linking CD3/TCR complexes on thymocytes from H-Y TCR transgenic (Tg) mice. In agreement with previous reports, we demonstrate that culturing DP thymocytes with plate-bound anti-TCR antibody induces downregulation of CD4 and CD8 and upregulation of CD69 expression. Nevertheless, the activated cells did not undergo apoptosis, as determined by viable cell recoveries and by quantitation of DNA fragmentation using the TUNEL assay. However, specific depletion of the DP subset occurred within 24 hr when thymocytes were incubated in the presence of both anti-TCR and the immunosuppressant cyclosporin A (CsA). CsA also induced depletion of anti-CD3 stimulated normal DP thymocytes. Using mice homozygous for the *lpr* or *gld* mutation, we also have shown that Fas/Fas ligand interactions are not involved in the CsA-induced death of TCR-stimulated DP thymocytes. These data verify that TCR cross-linking alone is insufficient to induce apoptosis of DP thymocytes and further suggest that TCR stimulation activates a CsA-sensitive protective pathway that interferes with signaling events leading to apoptosis in DP thymocytes.


*Developmental Immunology*, 1996, Vol 5, pp. 1–15
Reprints available directly from the publisher
Photocopying permitted by license only

© 1996 OPA (Overseas Publishers Association)
Amsterdam B.V. Published in The Netherlands
by Harwood Academic Publishers GmbH
Printed in Singapore

### Cross-Linking the TCR Complex Induces Apoptosis in CD4⁺8⁺ Thymocytes in the Presence of Cyclosporin A

HEATHER L. POETSCHKE, DAVID B. KLUG, DAWN WALKER, and ELLEN R. RICHIE*

*The University of Texas, M.D. Anderson Cancer Center, Science Park-Research Division, P.O. Box 389, Smithville, Texas 78957, USA*

Although it is generally agreed that TCR ligation is a minimal requirement for negative selection in the $CD4^+8^+$ double-positive (DP) thymocyte subset, the costimulatory requirements and specific signaling events necessary to induce apoptosis are not well defined. We have explored the consequences of cross-linking CD3/TCR complexes on thymocytes from H-Y TCR transgenic (Tg) mice. In agreement with previous reports, we demonstrate that culturing DP thymocytes with plate-bound anti-TCR antibody induces downregulation of CD4 and CD8 and upregulation of CD69 expression. Nevertheless, the activated cells did not undergo apoptosis, as determined by viable cell recoveries and by quantitation of DNA fragmentation using the TUNEL assay. However, specific depletion of the DP subset occurred within 24 hr when thymocytes were incubated in the presence of both anti-TCR and the immunosuppressant cyclosporin A (CsA). CsA also induced depletion of anti-CD3 stimulated normal DP thymocytes. Using mice homozygous for the *lpr* or *gld* mutation, we also have shown that Fas/Fas ligand interactions are not involved in the CsA-induced death of TCR-stimulated DP thymocytes. These data verify that TCR cross-linking alone is insufficient to induce apoptosis of DP thymocytes and further suggest that TCR stimulation activates a CsA-sensitive protective pathway that interferes with signaling events leading to apoptosis in DP thymocytes.

KEYWORDS: Apoptosis, cyclosporin A, negative selection, thymocytes.

### INTRODUCTION

Intrathymic T-cell development is the consequence of an orderly series of molecular events initiated by contact between bone marrow derived precursors and thymic stromal cells. Discrete stages of the T-cell developmental process are defined phenotypically by expression of CD4 and CD8 coreceptors and the TCR/CD3 complex. Productive rearrangement of the TCRβ chain locus is a prerequisite for transition of immature $CD4^-8^-$ double-negative (DN) cells to the $CD4^+8^+$ double-positive (DP) stage via a $CD4^-8^{lo}$ intermediate (Nikolic-Zugic and Bevan, 1988; Mombaerts et al., 1992; Dudley et al., 1994). Subsequent TCRα gene rearrangement within the DP subset results in low-level expression of αβTCR/CD3 complexes (Kearse et al., 1994). The process of thymic selection operates on these $TCR^{lo}$ DP cells to shape the final MHC-restricted TCR repertoire (Robey and Fowlkes, 1994). Positive selection refers to the maturation of thymocytes expression TCRs that mediate low-affinity interactions with self-peptides presented by self-MHC molecules (Ashton-Rickardt et al., 1994; Hogquist et al., 1994; von Boehmer, 1994). The minor fraction of DP thymocytes that undergoes positive selection differentiates to either $CD4^-8^+$ (MHC class I restricted) or $CD4^+8^-$ (MHC class II restricted) mature single-positive (SP) cells that express high levels of TCR/CD3 complexes. In contrast, DP thymocytes expressing TCRs with high affinity for self-MHC/self-peptide complexes undergo clonal deletion, a process referred to as negative selection (Fowlkes et al., 1988; Kisielow et al., 1988; Ashton-Rickardt et al., 1994). Because most DP thymocytes fail to express TCRs that interact with self-MHC molecules, they are neither positively nor negatively selected. These "neglected" DP thymocytes have a short life span (~3.5 days) *in vivo* and undergo apoptosis when cultured *in vitro* (Robey and Fowlkes, 1994; Kishimoto et al., 1995). Recently, direct evidence for

*Corresponding author.

thymocyte apoptosis *in situ* was obtained using a highly sensitive technique to detect DNA strand breaks, i.e., terminal deoxynucelotidyl transferase (TdT)-mediated labeling of nicked DNA with dUTP biotin (TUNEL) (Surh and Sprent, 1994).

Although negatively selected and neglected thymocytes undergo internucleosomal DNA fragmentation and morphological changes consistent with apoptosis, the signal transduction pathways and the role of APCs in promoting apoptosis are not yet resolved. Several groups have demonstrated that DP thymocytes expressing a transgenic TCR are depleted when cultured in the presence of antigen and APCs expressing appropriate MHC molecules (Swat et al., 1991; Iwabuchi et al., 1992; Vasquez et al., 1992). Cellular depletion also has been induced by antibody-mediated ligation of the TCR/CD3 complex. Anti-CD3 administered *in vivo* or in fetal thymic organ cultures results in thymocyte death associated with DNA degradation (Smith et al., 1989; Shi et al., 1991). However, there are conflicting reports concerning the requirement for APCs in anti-CD3-induced apoptosis *in vitro*. Although some studies conclude that antibody-mediated TCR cross-linking alone is sufficient to stimulate DNA fragmentation and/or cell death (McConkey et al., 1989a; Carlow et al., 1992; Migita et al., 1994), other groups report that anti-CD3-stimulated DP thymocytes fail to undergo apoptosis in the absence of APCs, suggesting that, in addition to TCR engagement, a second signal is required to induce apoptosis in the DP subset (Page et al., 1993; Punt et al., 1994; Kearse et al., 1995).

Regardless of whether one or two signals are required, it is generally agreed that negative selection and anti-CD3-induced depletion of DP thymocytes are active processes triggered by an endogenous suicide mechanism. The apoptotic response of DP thymocytes is induced by lower affinity TCR-peptide/MHC interactions than are required to induce proliferation of mature T cells (Vasquez et al., 1994). Nevertheless, tyrosine kinase activation, elevated intracellular calcium levels, and PKC activation have been found necessary to achieve anti-TCR or peptide/APC-induced apoptosis of DP thymocytes (McConkey et al., 1994; Migita et al., 1994; Vasquez et al., 1994). Even in the absence of biochemical or morphological evidence of apoptosis, triggering DP thymocytes through the CD3/TCR complex induces phenotypic changes consistent with cellular activation. Thus, TCR ligation effectively reduces CD4 and CD8 levels while increasing CD5 and CD69 expression even when apoptosis is not a consequence of cross-linking the TCR complex (Page et al., 1993; Swat et al., 1993; Kearse et al., 1995). These findings suggest that a TCR-mediated signal transduction pathway is operative in DP thymocytes regardless of whether the signal culminates in DNA fragmentation and cell death.

In the present study, we utilized transgenic mice expressing a TCR that is specific for the H-Y male antigen presented by H-$2^b$ class I molecules to further explore signal requirements for apoptosis in DP thymocytes (Kisielow et al., 1988). The fraction of transgenic $CD4^+8^+$ cells containing fragmented DNA after incubation with anti-TCR$\alpha$ chain antibody was quantitated using the TUNEL assay (Gorczyca et al., 1993; Kishimoto et al., 1995). The data demonstrate that TCR ligation induces downregulation of CD4 and CD8 as well as upregulation CD69 expression in the absence of significant DNA fragmentation. However, the addition of cyclosporin A (CsA) rapidly induces DNA fragmentation and depletion of TCR-stimulated DP thymocytes. We suggest that either CsA provides a second signal to induce apoptosis in activated DP thymocytes or that TCR signaling induces a CsA-sensitive protective pathway that interferes with the manifestation of apoptosis in the activated DP population. Prior activation of the cells via *in vivo* engagement of the transgenic TCR with self-MHC molecules is not necessary because CsA depletes anti-TCR-activated transgenic DP thymocytes from a nonselecting background as well as normal DP thymocytes activated by anti-CD3 cross-linking. Furthermore, similar experiments carried out with *lpr* and *gld* mutant mice demonstrate that Fas/FasL interactions are not responsible for CsA-dependent depletion of TCR-activated DP thymocytes.

### RESULTS

### Engagement of the TCR Induces Phenotypic Changes but not DNA Fragmentation in DP Transgenic Thymocytes

Thymocytes from female H-$2^b$ H-Y transgenic mice were cultured for various intervals in the presence or absence of plate-bound anti-TCR$\alpha$ chain antibody (T3.70) to determine whether signaling through the TCR alone could induce phenotypic alterations and/or apoptosis. TCR ligation did not result in significant thymocyte depletion at 24 hr. In seven experiments, the recovery of viable cells after incubation in antibody-coated wells ranged from 72 to 100% of the cells obtained following incubation in medium alone. Furthermore, there was no difference in the percentage of antibody treated versus untreated cells falling within a viable cell gate defined by forward and 90° light-scattering properties. To determine the effect of TCR cross-linking on surface marker expression, immunofluorescence (IF) profiles were obtained, and the results of a representative experiment are shown in Fig. 1A. Consistent with previous reports (Page et al., 1993; Kearse et al., 1995), TCR ligation induced a marked decrease in CD4 and CD8 expression on the DP subset. CD4 and CD8 downregulation was detectable as early as 6 hr and expression continued to decrease at later time points. To further characterize the effect of TCR ligation on the DP subset, the expression of CD69, an early activation marker, was evaluated on DP cells defined by the electronic gate shown in Fig. 1A. CD69 surface expression was upregulated by anti-TCR stimulation within 6 hr. By 24 hr >70% of the DP subset expressed CD69, suggesting that cross-linking the CD3/TCR complex transduces activation signals in the majority of DP thymocytes.

Although there is general agreement that stimulation of DP thymocytes with antigen and APCs induces DNA fragmentation and apoptosis, the consequences of antibody-mediated ligation with anti-TCR antibody in the absence of APCs are controversial (McConkey et al., 1989a; Carlow et al., 1992; Page et al., 1993; Migita et al., 1994; Punt et al., 1994; Kearse et al., 1995). To further explore this issue, we employed the TUNEL assay, which has been shown to be more sensitive than other methods for detection of DNA fragmentation (Kishimoto et al., 1995). Thymocytes recovered 24 hr after incubation in the presence or absence of T3.70 antibody were stained for CD4 and CD8 surface markers and subsequently fixed for nucleotide incorporation in the presence (or absence) of TdT. The data in Fig. 1B reflect b-dUTP incorporation in DP thymocytes using the same gates for $CD4^+8^+$ cells shown in Fig. 1A. By 24 hr, after incubation in medium alone, 27% of the control cells incorporated b-dUTP, a finding consistent with the report of Kishimoto and colleagues in which the TUNEL method was used to quantitate apoptosis in nonstimulated thymocyte cultures (Kishimoto et al., 1995). Similarly, 24% of the DP thymocytes incorporated b-dUTP after incubation in the presence of plate-bound T3.70 antibody. Taken together, the data in Fig. 1 suggest that although DP thymocytes are signaled via TCR ligation to express an altered profile of cell-surface markers, this activation pathway does not extend to induction of DNA fragmentation, a hallmark of apoptosis.

### CsA Inhibits CD69 Expression and Induces DNA Fragmentation and Cellular Depletion in TCR-Stimulated Transgenic DP Thymocytes

CsA is an immunosuppressive drug that inhibits mature T-cell activation (Schreiber and Crabtree, 1992). CsA is thought to act by binding to its cytosolic receptor, cyclophilin, to form a complex that inhibits the activation of the calcium- and calmodulin-dependent serine/threonine phosphatase, calcineurin. To determine if CsA affects T3.70-mediated activation of DP thymocytes, $H\text{-}2^b$ H-Y transgenic thymocytes were incubated in the presence or absence of plate-bound T3.70 antibody, and 100 ng/ml of CsA was added to selected cultures at initiation. In preliminary titration experiments, concentrations of CsA ranging from 10 ng/ml to 1 µg/ml gave similar results in the presence or absence of antibody. Fig. 2 shows surface marker expression on thymocytes recovered at various intervals. The addition of CsA had no effect on DP thymocytes incubated in the absence of T3.70 antibody. However, inclusion of CsA in T3.70-stimulated cultures resulted in a dramatic decrease in the percentage of DP cells recovered at 24 hr. In addition, CsA inhibited the appearance of $CD4^{lo}8^{lo}$ cells and induction of CD69 expression, phenotypic alterations that were consistently observed after TCR cross-linking in the absence of CsA. These data suggest that CsA effectively depletes TCR-stimulated DP thymocytes. Table 1 presents the percentage and absolute number of thymocytes recovered in each of the four major thymocyte $CD4^+8^+$ subsets after incubation in the presence or absence of plate-bound antibody and CsA. Although the decreased percentage of DP cells is partially compensated for by an increased percentage of cells in the DN and SP compartments, the absolute number of cells recovered in each subset shows that cellular depletion is restricted primarily to the DP subset. The absence of a $CD4^{lo}8^{lo}$ population may, as Kearse and colleagues assert, indicate that CsA inhibits downregulation prior to CsA-induced DP death or rapid depletion following coreceptor downregulation.

To determine if the depletion of activated DP thymocytes was a consequence of apoptosis, the

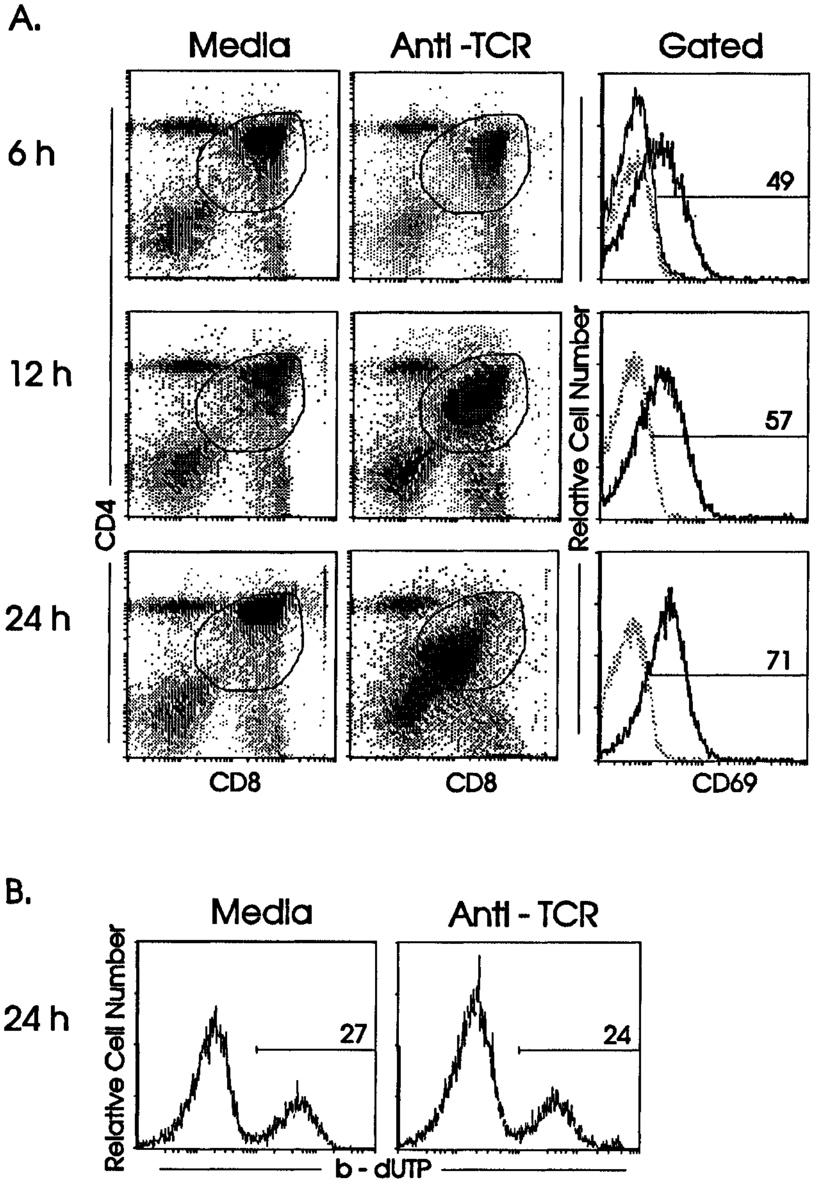

FIGURE 1. Antibody-mediated cross-linking of the TCR complex on DP thymocytes induces phenotypic alterations in the absence of DNA fragmentation. Thymocytes from female H-$2^b$ H-Y TCR transgenic mice were incubated with plate-bound T3.70 antibody for the indicated time intervals. (A) Three-color IF analysis of CD4, CD8, and CD69 expression. $CD4^+8^+$ cells falling within the indicated electronic gates were assessed for CD69 expression. The histogram shows CD69 expression on DP cells incubated in media (broken line) or with anti-TCR antibody (dark solid line). The lighter solid line shown only in the uppermost histogram represents CD69 expression on DP thymocytes prior to culture. The percentage of CD69-positive cells induced by anti-TCR stimulation are shown. (B) DNA fragmentation was determined by the percentage of cells that incorporated b-dUTP in the TUNEL assay performed in the presence of TdT. Controls stained in the absence of TdT showed <2% b-dUTP incorporation (data not shown). The percentage of TUNEL-positive cells after 24 hr incubation are shown.

TABLE 1
Recovery of Thymocyte Subsets after Anti-CD3-Stimulation in the Presence or Absence of CsA[a]

| | Ab | CsA | Number ($\times 10^{-5}$) | | | | Percentage | | | |
|---|---|---|---|---|---|---|---|---|---|---|
| | | | DN | DP | CD4+ | CD8+ | DN | DP | CD4+ | CD8+ |
| Exp. 1 | – | – | 2.1 | 8.7 | 0.8 | 0.4 | 16 | 65 | 6 | 3 |
| | – | + | 2.8 | 11 | 1.2 | 0.6 | 17 | 65 | 7 | 4 |
| | + | – | 2.0 | 8.9 | 0.6 | 0.3 | 16 | 72 | 5 | 2 |
| | + | + | 1.6 | 1.6 | 0.6 | 0.2 | 36 | 37 | 13 | 4 |
| Exp. 2 | – | – | ND[b] | ND | ND | ND | ND | ND | ND | ND |
| | – | + | 1.3 | 12 | 0.8 | 1.0 | 8 | 77 | 5 | 6 |
| | + | – | 1.7 | 10 | 0.6 | 1.0 | 12 | 74 | 4 | 7 |
| | + | + | 3.3 | 1.7 | 1.4 | 4.0 | 45 | 23 | 19 | 6 |

[a] After 24 hr, the number of viable cells was determined by trypan blue dye exclusion. CD4/8 subset distribution was determined on cells that were stained with CD4-PE and CD8-FITC. The number of cells in each subset was calculated by multiplying the total number of viable cells recovered by the percentage of cells in each phenotypic subset.
[b] ND = not determined.

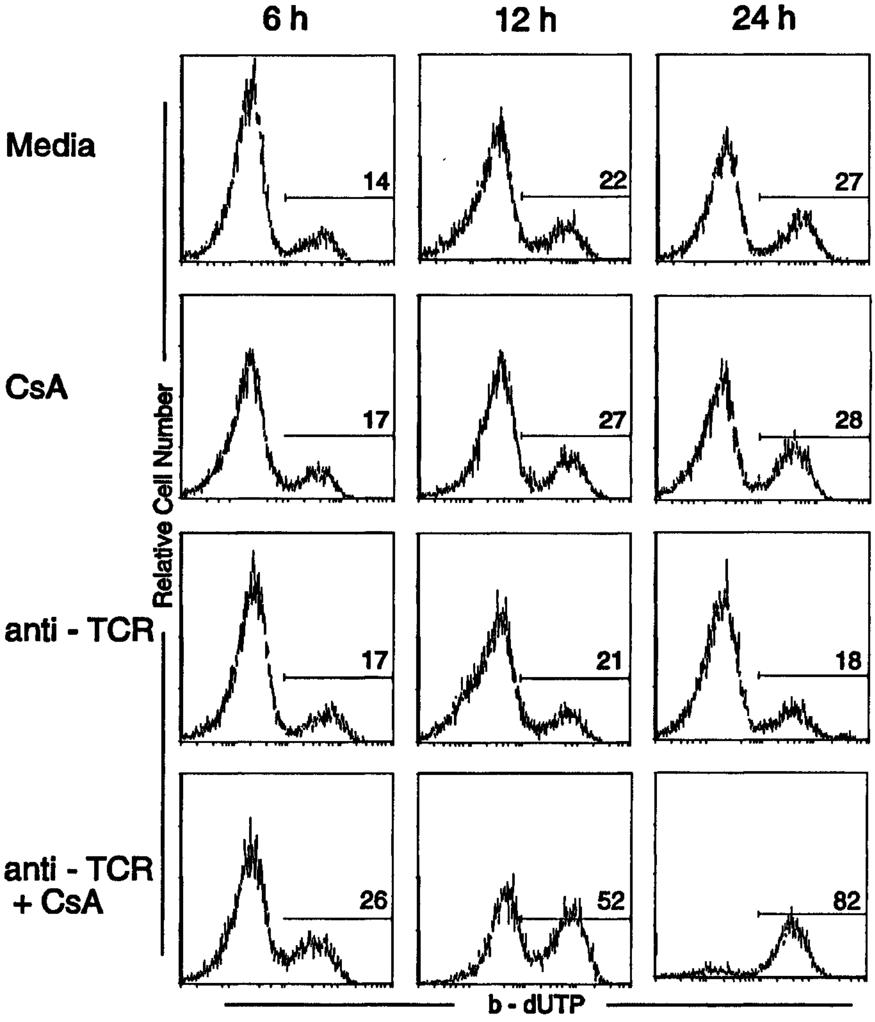

FIGURE 3. CsA induces apoptosis in anti-TCR-stimulated DP thymocytes from female H-2[b] H-Y TCR transgenic mice. Thymocytes from female H-2[b] H-Y TCR transgenic mice were incubated for the indicated intervals in the presence or absence of plate-bound T3.70 antibody. CsA (100 ng/ml) was added as indicated to selected cultures. TUNEL staining of electronically gated DP cells was determined by b-dUTP incorporation in the presence of TdT.

TUNEL assay was performed. The data in Fig. 3 show that as expected, cells incubated in the presence of T3.70 or CsA alone did not show a substantial increase in b-dUTP incorporation above that observed in media controls. In contrast, b-dUTP incorporation was substantially increased by 6 to 12 hr in DP thymocytes cultured in the presence of both T3.70 and CsA, and by 24 hr, >80% of the remaining DP cells contained fragmented DNA. Notably, CsA failed to cause DNA fragmentation in the T3.70 or anti-CD3-stimulated DN and SP subsets (data not shown). These data demonstrate that DP thymocytes from H-Y TCR transgenic H-$2^b$ mice undergo apoptosis when incubated in the presence of plate-bound anti-TCR antibody and the immunosuppressant CsA.

### H-$2^d$ H-Y Transgenic and Normal DP Thymocytes Undergo Apoptosis after Stimulation via the TCR/CD3 Complex in the Presence of CsA

The previous experiments were carried out with female H-$2^b$ H-Y transgenic mice in which developing thymocytes are subject to positive selection. To address the possibility that the consequences of *in vitro* TCR ligation were due, in part, to previous engagement between the transgenic TCR and selecting H-$2^b$ molecules *in vivo*, similar studies were carried out with H-$2^d$ H-Y transgenic mice in which positive selection of the H-Y TCR does not occur because the appropriate restricting MHC allele is not present. As in the H-$2^b$ background, DP thymocytes from H-$2^d$ transgenics respond to TCR ligation by downmodulating CD4 and CD8 surface levels and inducing CD69 expression (Table 2 and data not shown). Although the H-$2^d$ transgenics incorporated high background levels of d-UTP, TCR ligation induced little, if any, DNA fragmentation above that observed in unstimulated cells. TCR ligation in the presence of CsA reduced the percentage and absolute number of H-$2^d$ transgenic DP cells, and the majority of DPs that were not deleted by 24 hr failed to express CD69 and contained fragmented DNA (Table 2 and data not shown). Likewise, DP thymocytes from normal C57Bl/6J mice were activated in response to anti-CD3-mediated TCR crosslinking, as revealed by downregulation of CD4 and CD8 and upregulation of CD69. The addition of CsA to antibody-stimulated cultures reduced the percentage of cells in the DP compartment, inhibited CD69 expression and augmented b-dUTP incorporation in the remaining DP cells (Table 2 and data not shown). Interestingly, the percentage of TUNEL positive cells is generally lower in normal versus H-$2^d$ H-Y transgenic thymocytes. This phenomenon was also observed when normal thymocytes were compared with H-$2^b$ transgenic thymocytes that had relatively low background levels of b-dUTP incorporation (Fig. 3). Although the basis for this distinction between normal and transgenic thymocytes requires further investigation, the results for H-$2^d$ transgenic and normal thymocytes demonstrate that alterations in surface marker phenotype and susceptibility to CsA-induced apoptosis are solely a consequence of TCR engagement *in vitro* and not a consequence of previous *in vivo* signal transduction events.

### CsA-Induced Apoptosis Is Not a Function of Fas/FasL Interactions

The Fas cell surface receptor, a member of the tumor-necrosis factor receptor family, induces apoptosis of

TABLE 2
Response of DP Thymocytes from H-Y H-$2^d$ Transgenic and Normal Mice to TCR Ligation in the Presence or Absence of CsA[a]

| | | Percentage of $CD69^+$ Cells | | | | Percentage of Cells Incorporating b-dUTP | | | |
|---|---|---|---|---|---|---|---|---|---|
| Cells | Exp. | Control | CsA | Ab | CsA+Ab | Control | CsA | Ab | CsA+Ab |
| H-$2^d$ H-Y Transgenic | 1 | 8 | 6 | 77 | 19 | ND | ND | ND | ND |
| | 2 | ND[b] | ND | ND | ND | 37 | 41 | 38 | 89 |
| | 3 | 5 | 4 | 64 | 14 | 30 | 46 | 35 | 81 |
| | 4 | 5 | 4 | 68 | 16 | 37 | 41 | 53 | 82 |
| Normal C57Bl/6 | 1 | 5 | 5 | 53 | 7 | ND | ND | ND | ND |
| | 2 | ND | ND | ND | ND | 22 | 30 | 29 | 51 |
| | 3 | ND | ND | ND | ND | 16 | 20 | 22 | 40 |
| | 4 | 5 | 5 | 65 | 21 | 18 | 20 | 22 | 41 |

[a] Thymocytes were incubated for 24 hr in the presence or absence of plate-bound anti-CD3. CsA (100 ng/ml) was added to selected cultures at initiation. The recovered cells were stained with anti-CD4-PE, anti-CD8-FITC, and either anti-CD69-biotin or fixed for the TUNEL assay, as described in Materials and Methods. CD69 expression and b-dUTP incorporation are presented for gated DP thymocytes.
[b] ND = not determined.

activated T cells upon interaction with its ligand, FasL. Recently, it was reported that DP thymocytes not only express Fas, but also undergo apoptosis in the presence of anti-Fas antibody (Ogasawara et al., 1995). We utilized the mutant *lpr* mouse strain, which is deficient in Fas expression (Watanabe-Fukkunaga et al., 1992), as well as the mutant *gld* (Takahashi et al., 1994) mouse strain, which is deficient in FasL expression, to investigate whether CsA-induced apoptosis of TCR-stimulated DP thymocytes is a function of Fas/FasL interaction. Both *lpr* and *gld* DP thymocytes are activated by Ab-mediated cross-linking of cell surface CD3 to downregulate CD4 and CD8 and upregulate CD69 (Fig. 4 and data not shown). In addition, activated *lpr* and *gld* DP thymocytes undergo apoptosis in the presence of CsA. The percentage of DPs containing fragmented DNA in the mutant strains is similar to that observed in thymocytes from normal mice. An earlier report showing that CsA inhibits activation-induced expression of FasL (Dhein et al., 1995) supports these findings and indicates that induction of FasL expression and interaction with the Fas receptor are not responsible for CsA-mediated apoptosis in TCR-activated thymocytes.

### Cycloheximide Inhibits Anti-TCR-Induced Phenotypic Alterations and CsA-Induced Apoptosis

Previous studies have shown that activation-induced cell death in thymocytes and T-cell hybridomas is an active process that is inhibited by CHX (McConkey et al., 1989b; Ucker et al., 1989; Vasquez et al., 1992, 1994). To determine if CsA-induced apoptosis of activated thymocytes is similarly dependent on protein synthesis, CHX was included in thymocyte cultures in the presence or absence of plate-bound anti-CD3 and CsA. The recovered cells were evaluated for surface marker expression and incorporation of b-dUTP in the TUNEL assay. There was no toxicity associated with the concentration of CHX (20 μg/ml) used in these experiments, although minimal toxicity was observed at higher concentrations (40 μg/ml). CHX alone affected neither the surface phenotype nor the percentage of TUNEL

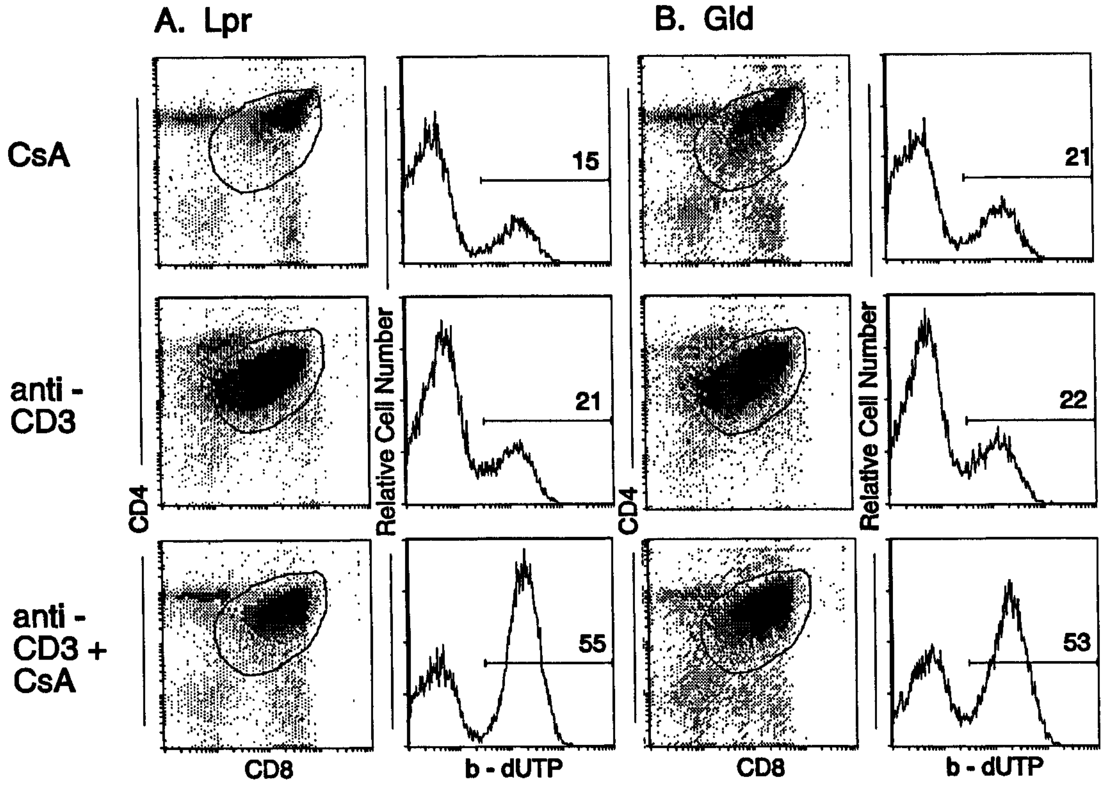

FIGURE 4. CsA-induced apoptosis of TCR-activated thymocytes is not a function of the Fas/FasL interaction. Thymocytes obtained from (A) *lpr* or (B) *gld* mice were stimulated with anti-CD3 for 24 hr in the presence or absence of CsA. In addition to assessment of CD4/8 profiles, DNA fragmentation in electronically gated DP cells was assessed by the percentage of cells that stained positive in the TUNEL assay.

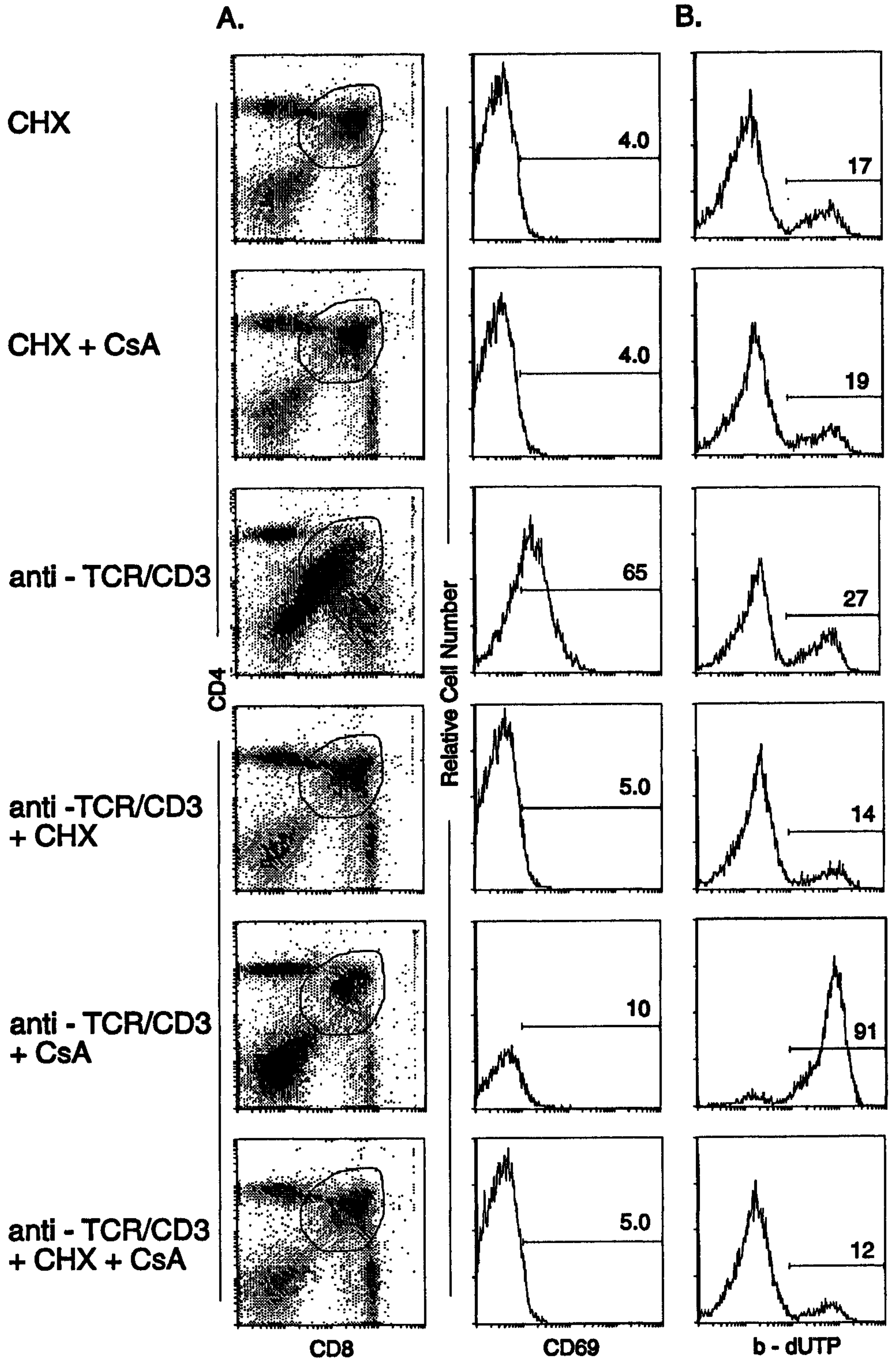

FIGURE 5. Effect of CHX on the surface marker alterations and DNA fragmentation induced by TCR ligation in the presence and absence of CsA. Thymocytes from female H-Y H-$2^b$ transgenics were cultured with plate-bound (A) T3.70 or (B) anti-CD3. CsA and/or CHX (20 μg/ml) were added to selected wells at initiation. CD69 expression and TUNEL reactivity were determined on the gated DP subset. The percentage of cells within the indicated DP gate was 66% with CHX, 67% with CHX + CsA, 49% with anti-TCR, 66% with anti-TCR + CHX, 20% with anti-TCR + CsA, and 66% with anti-TCR + CHX + CsA.

positive thymocytes. However, the data in Fig. 5 show that CHX completely abrogated antibody-induced upregulation of CD69 on the DP subset. Interestingly, CHX treatment also prevented the downregulation of CD4 and CD8 that characteristically accompanies TCR ligation of DP cells. Similar results were obtained with 10 µg/ml of CHX (data not shown). Furthermore, CHX treatment profoundly inhibited CsA-induced apoptosis of TCR-activated DP thymocytes. When CHX was also included in cultures containing anti-TCR plus CsA, there was no reduction in the percentage of cells in the DP compartment. In addition, the DP cells obtained from these cultures failed to incorporate b-dUTP beyond control levels. Taken together, the data demonstrate that anti-CD3-induced CD4 and CD8 downregulation as well as CD69 upregulation require active protein synthesis as does the apoptotic response that occurs when DP thymocytes are stimulated with anti-TCR in the presence of CsA.

### DISCUSSION

The present investigation has examined the consequences of cross-linking the TCR complex on DP thymocytes from transgenic and normal mice. We found that antibody-mediated TCR ligation results in downregulation of CD4 and CD8 levels as well as induction of CD69 expression but stimulates minimal, if any, DNA fragmentation or cellular depletion above that observed in unstimulated cells. Nevertheless, stimulation via the TCR activates a potentially apoptotic pathway in DP thymocytes because the addition of CsA to anti-TCR-activated thymocytes induces rapid apoptosis detected by DNA fragmentation and cellular depletion. Thymocytes that are incubated with CsA in the absence of anti-TCR antibody do not undergo cellular activation or apoptosis. Therefore, CsA-mediated induction of apoptosis in the DP subset is dependent upon signal transduction events that result from cross-linking the TCR complex.

There have been conflicting reports concerning the effect of CsA on negative selection *in vivo* and thymocyte deletion *in vitro*. Similar to the results presented here, two other groups have shown that CsA augments the deletion of thymocytes activated *in vitro* by anti-CD3 or phorbol ester plus ionophore (McCarthy et al., 1992; Cairns et al., 1993; Curnow and Schmitt-Verhulst, 1994). However, in apparent conflict with these results, *in vivo* administration of CsA is reported to inhibit depletion of normal thymocytes that express self-reactive TCRs (Gao et al., 1988; Jenkins et al., 1988) and to delay negative selection in H-Y TCR transgenic H-2$^b$ male mice (Urdahl et al., 1994). This apparent discrepancy may be due to the fact that CsA not only adversely affects lymphocyte activation, but also inhibits accessory cell function. In this regard, CsA treatment of splenic APCs inhibits their costimulatory activity *in vitro* (Cairns et al., 1993), and ultrastructural studies show that CsA damages thymic reticuloepithelial cells *in vivo* (Fabien et al., 1992). Therefore, CsA-mediated blockade of negative selection *in vivo* may be an indirect consequence of interference with the costimulatory activity of thymic APCs. Another factor that is likely to affect the outcome of CsA treatment and to reconcile some of the contradictory findings is the maturation stage of the target cells. For example, the addition of CsA to mature T-cell hybridomas has been repeatedly shown to block DNA fragmentation *in vitro* (Shi et al., 1989; Zacharchuck et al., 1990). In contrast, other studies have reported that CsA fails to inhibit TCR-mediated activation-induced apoptosis in cultures of immature DP thymocytes (Anderson et al., 1995; Wang et al., 1995). It seems likely that differences in the signaling pathways activated by TCR ligation in mature versus immature T cells contribute to the opposing conclusions reached in prior reports concerning the effect of CsA on activation-induced apoptosis. Finally, it should be emphasized that because apoptosis is maximally induced when TCR-activated thymocytes are incubated in the presence of costimulatory signals (Iwabuchi et al., 1992; Page et al., 1993; Punt et al., 1994), the potentiating effect of CsA on DNA fragmentation is not apparent under these conditions. Wang and colleagues reported that although CsA inhibits positive selection, it fails to inhibit DNA fragmentation induced in thymocytes by anti-TCR or superantigen in the presence of accessory cells (Wang et al., 1995). This conclusion does not conflict with our data showing that in the absence of costimulation, CsA potentiates apoptosis in TCR-activated thymocytes.

There are at least two possible interpretations of the results obtained in this investigation: (1) TCR ligation not only triggers molecular events that can culminate in apoptosis, but also induces a CsA-sensitive pathway capable of blocking activation-induced DNA fragmentation and cell death; and (2) CsA provides a second signal that in combination with signals generated by TCR cross-linking results in apoptosis. In this first scenario, TCR-mediated activation of DP cells in the absence of CsA would result in molecular events that confer protection against a TCR-induced cellular suicide process. CsA treatment would inhibit an essential component of the protective pathway, thereby permitting the cells to undergo activation-induced apoptosis. In the second scenario, a signal transduction process initiated by the TCR would combine with a separate CsA-derived signal to induce apoptosis. Although the present data do not distinguish between these two possibilities, we favor the former explanation because CsA is known to inhibit T cell activation events. CsA exerts its immunosuppressive activity by inactivating calcineurin, a calcium- and calmodulin-dependent serine/threonine phosphatase (Schreiber and Crabtree, 1992). Calcineurin activity is required for activation of NF-AT and expression of *c-rel*, two DNA-binding proteins that regulate transcription of IL-2 and IL-2-receptor α-chain genes, respectively (McCaffrey et al., 1993; Venkataraman et al., 1995). Perhaps calcineurin activates transcription factors that regulate genes, encoding proteins that inhibit DNA fragmentation, thereby protecting activated DP thymocytes from undergoing apoptosis. Inactivation of calcineurin by CsA could result in abrogation of this protective pathway, permitting apoptosis to proceed. Alternatively, calcineurin is also known to affect other targets including substrates of cAMP-dependent kinases (Cohen, 1989).

Previous reports have shown that stimulation of normal thymocytes with PMA or calcium ionophore induces DNA fragmentation, whereas costimulation with appropriate concentrations of both reagents fails to do so (Kizaki et al., 1989; McCarthy et al., 1992; Cairns et al., 1993) The addition of CsA to PMA plus ionophore-treated thymocytes reverses the protective effect, resulting in increased DNA fragmentation (McCarthy et al., 1992; Cairns et al., 1993). These observations suggest that apoptosis is a potential consequence of either PKC activation or increased intracellular calcium concentration, but that DNA fragmentation is inhibited by an active process induced by a combination of PKC and calcium-mediated signals. Although the effect of TCR ligation on surface marker expression was not determined, the same report showed that CsA-induced apoptosis induced in thymocytes incubated with plate-bound anti-CD3 antibodies (Cairns et al., 1993). However, the extent of DNA fragmentation was low compared to our data, an inconsistency that is likely due to the greater sensitivity of the TUNEL assay used in the present investigation. Based on the current work and previous reports cited above, we suggest that triggering DP thymocytes via TCR ligation activates a signal transduction pathway(s) similar to that induced by coincubation with PMA and ionophore, implying that both PKC and calcium play a role in the anti-CD3-induced pathway that prevents manifestation of activation-induced apoptosis. Furthermore, the apoptotic pathway that is stimulated in DP thymocytes by TCR ligation in the presence of CsA requires protein synthesis, as demonstrated by the finding that CHX effectively blocks DNA fragmentation.

In keeping with the notion that mature and immature thymocytes are distinguished in part by variations in signal transduction circuitry, it is interesting to note that, as in the DP subset, CsA prevented anti-TCR-induced CD69 expression on mature $CD4^{-}8^{+}$ thymocytes from H-Y transgenic mice (data not shown). Nevertheless, in contrast to the DP subset, the $CD4^{-}8^{+}$ subset was resistant to apoptosis induced by stimulation via the TCR in the presence of CsA. Similar results were obtained for normal SP thymocytes stimulated with anti-CD3 in the presence or absence of CsA. These data suggest that as thymocytes undergo intrathymic maturation, TCR ligation fails to trigger the same apoptotic pathway that is induced in the DP subset or that the protective factor(s) preventing TCR-stimulated apoptosis is constitutively produced and not subject to CsA-mediated repression. In this regard, it will be interesting to determine if members of the *bcl*-2 family play a role in this process because *bcl*-2 has been shown to inhibit apoptosis induced in thymocytes by a variety of agents, including radiation, glucocorticoids, and *in vivo* injection of anti-CD3 (Sentman et al., 1991; Strasser et al., 1991; Siegel et al., 1992). Another molecular mechanism that is associated with activation-induced cell death in thymocytes is the signal transduction pathway induced by Fas/FasL interaction. Since anti-Fas antibody induces DNA fragmentation and cell death in DP thymocytes (Ogasawara et al., 1995), it was of interest to determine if anti-TCR plus CsA-induced apoptosis in DP thymocytes was a function of signals transduced via the Fas receptor. The data showing that CsA induces DNA fragmentation in anti-CD3-stimulated *lpr* and *gld* DP thymocytes clearly demonstrate that Fas/FasL interaction does not play a role in the CsA-induced apoptosis of activated DP thymocytes. However, the possibility remains that CsA-induced death of TCR-activated DP cells mimics the signal transduction events downstream of the Fas/FasL interaction.

Cross-linking the TCR complex by plate-bound antibody induced CD69 expression on the majority of TCR transgenic DP thymocytes from nonselecting H-$2^d$ mice as well as on DP thymocytes from normal mice, suggesting that in contrast to a previous interpretation (Swat et al., 1993), the TCR-activation pathway is coupled to inducibility of CD69 expression prior to positive selection. The present data also demonstrate that upregulation of CD69 expression and downregulation of CD4 and CD8 expression are achieved by TCR ligation in the absence of additional signaling events. However, there is continuing controversy concerning the issue of whether a second signal, in addition to TCR cross-linking, is required for induction of apoptosis in DP thymocytes (McConkey et al., 1989a; Carlow et al., 1992; Page et al., 1993; Migita et al., 1994; Punt et al., 1994; Kearse et al., 1995). The present data support the notion that TCR ligation alone is insufficient to stimulate activation-induced DNA fragmentation in DP thymocytes. APCs have been shown to effectively provide costimulatory signals that induce deletion of antigen or anti-TCR-activated thymocytes (Iwabuchi et al., 1992; Page et al., 1993). Costimulation is thought to be supplied by interaction between APC surface ligands and corresponding receptors expressed on activated thymocytes. We demonstrate in this report that CsA can efficiently replace the requirement for APCs in achieving DNA fragmentation in anti-TCR-stimulated DP thymocytes. It is possible that APCs and CsA similarly affect signaling pathways that result in apoptosis. If so, then the function of APCs may not be to provide a second signal that generates novel molecular events required for apoptosis, but instead to provide a second signal that interferes with a TCR-activated protective pathway that prevents activation-induced apoptosis.

Although both normal and transgenic thymocytes are susceptible to apoptosis induced by stimulation with plate-bound anti-TCR in the presence of CsA, there was a consistent difference in the percentage of DP cells containing fragmented DNA 24 hr after culture initiation. The TCR transgenic DP thymocytes were almost uniformly TUNEL positive, whereas a significant fraction of normal DP thymocytes remained TUNEL negative. This phenomenon was observed regardless of whether the transgenic thymocytes were obtained from a selecting or nonselecting MHC haplotype. The nature of the resistant DP subset from normal mice was not determined, but we surmise that these cells may be defective in TCR-mediated signal transduction because they (1) represent cells that have not yet entered the apoptotic pathway (delayed kinetics), and/or (2) represent early DPs that express low levels of CD3/TCR complexes precluding efficient signal transduction, and/or (3) represent cells that have not yet coupled surface CD3/TCR complexes to appropriate intracellular signaling pathways. It was not possible to adequately assess the first possibility because initial attempts to culture normal thymocytes for 48 hr yielded a high level of nonspecific cell death in the absence of anti-CD3. To address the second possibility, we evaluated the response of DP thymocytes from mice homozygous for a mutant TCR$\alpha$ gene (TCR$\alpha^-/^-$) because these cells express extremely low levels of cell surface CD3 (Mombaerts et al., 1992; Philpott et al., 1992). Cross-linking CD3 complexes with plate-bound anti-CD3 not only fails to induce characteristic phenotypic changes in the TCR$\alpha^-/^-$ DP thymocytes, but also fails to induce DNA fragmentation in the presence of CsA (data not shown). However, it is not possible to distinguish if the lack of responsiveness to anti-CD3 cross-linking is due to low levels of CD3 expression and/or to differences in the composition of CD3 complexes on TCR$\alpha^-/^-$ compared to normal thymocytes. Therefore, further studies are required to define the nature of the TUNEL negative normal DP thymocytes that resist anti-CD3 plus CsA-induced apoptosis.

In summary, this report verifies that although antibody-mediated cross-linking of the CD3/TCR complex on DP thymocytes activates a signal transduction pathway resulting in altered surface marker expression, TCR ligation alone is insufficient to induce apoptosis in the DP subset. However, H-Y TCR transgenic as well as normal DP thymocytes are subject to rapid apoptosis when CD3/TCR complexes are cross-linked in the presence of CsA. These data suggest the existence of a CsA-sensitive protective pathway that prevents TCR-stimulated DNA fragmentation. By using the experimental model described in this report, it is now possible to investigate specific components of the signal transduction pathway(s) that alter expression of differentiation antigens and that expedite or inhibit activation-induced cell death in DP thymocytes.

### MATERIALS AND METHODS

### Mice

Breeding pairs of H-2$^b$ H-Y TCR transgenic mice (Kisielow et al., 1988) and H-2$^d$ H-Y TCR transgenic mice were kindly supplied by Dr. Dan Littman (Department of Microbiology and Immunology, University of California – San Francisco) and Dr B.J. Fowlkes (National Institutes of Health, Bethesda, MD), respectively. C57BL/6J, TCRα($^-$/$^-$), *lpr* and *gld* mice were purchased from Jackson Laboratories (Bar Harbor, ME). All mice were bred and/or maintained in accordance with institutional guidelines in the animal facility at the M.D. Anderson Science Park Research Division.

### Preparation and Culture of Thymocytes

Single-cell suspensions were prepared from thymuses of 4–8 week-old mice by pressing the tissue through a nylon mesh into cold 5 mM HEPES-buffered Hank's balanced salt solution (HBSS) containing 1% bovine serum albumin, pH 7.4. After washing, the cells were resuspended in RPMI 1640 media supplemented with 55 µM 2-ME, 2 mM L-glutamine, 1 mM sodium pyruvate, 1 mM non-essential amino acids (Gibco BRL, Grand Island, NY), and 10% FCS (Summit Biotechnology, Greeley, CO), and distributed at 1–2.5 × $10^6$/ml in 24-well tissue-culture plates. The wells were pretreated with HBSS buffer or 10–20 µg of appropriate mAb for 3 hr or overnight at 37°C and washed three times with buffer before use. After incubation at 37°C, 5% $CO_2$ for various intervals, cells were recovered by washing the wells three times with HBSS buffer. Cyclosporin A (CsA), (Sandoz, East Hanover, NJ) was used at a final concentration of 100 ng/ml. Cycloheximide (CHX) (Sigma, St. Louis, MO) was used at a concentration of 20 µg/ml.

### Antibodies, Immunofluorescence, and Flow Cytometry

PE-conjugated anti-CD4 (clone RM4-5), FITC-conjugated anti-CD8 (clone 53–6.7), and biotinylated anti-CD69 (clone H1.2F3) were obtained from PharMingen (San Diego, CA). Anti-CD3ε (500A) and anti-Vα3 (T3.70) were purified from hybridoma culture supernatant using protein A or protein G columns. Cells for three-color immunofluorescence analysis were suspended in HBSS containing 1% BSA and 0.1% sodium azide and incubated with directly conjugated antibodies on ice for 30 min followed by three washes to remove excess reagents. To detect binding of biotinylated antibody, the cells were incubated with allophycocyanin-conjugated strepavidin (APC-SA) (Molecular Probes, Eugene, OR) for 15 min on ice followed by additional washes and fixation in 1% paraformaldehyde. Stained cells were analyzed with a Coulter Epics Elite flow cytometer (Miami, FL) equipped with an argon laser (488 nm) for FITC and PE excitation and a helium neon laser (633 nm) for APC-SA excitation. Data were collected on 10–20 × $10^3$ viable cells using a four-decade log amplifier and stored in list mode for subsequent analysis using Coulter Elite software. Immunofluorescence and TUNEL profiles were restricted to cells that fell within a viable gate established by forward and 90° light-scatter profiles.

### TUNEL Assay

The terminal dexoynucleotidyl transferase (TdT)-mediated dUTP-biotin nick end labeling (TUNEL assay) reaction was performed according to Kishimoto et al. 1995 with certain modifications. Thymocytes that had been surface stained for CD4 and CD8 were fixed in 1% paraformaldehyde followed by 70% ethanol at 4°C for 15 min each. After two washes in HBSS, the pellet was resuspended in TdT buffer (200 mM potassium cacodylate, 25 mM Tris-HCl, 250 µg/ml BSA) plus 2.5 mM cobalt chloride, 0.02 nM biotin-16-dUTP (b-dUTP), and 0.5 U/µl TdT (Boehringer Mannhein, Indianapolis, IN). Cells were incubated in the absence of TdT as a negative control. After incubation at 37°C for 30 min, the cells were washed twice with HBSS containing 1% BSA and 0.1% sodium azide. TdT-dependent incorporation of dUTP-biotin was visualized by APC-SA staining for 30 min on ice.

### ACKNOWLEDGMENTS

H.L. Poetschke and D.B. Klug contributed equally to this work. The authors thank Dennis Walker for performing the flow cytometric analyses and Carrie McKinley for help in manuscript preparation. This research was supported by National Institutes of Health Grant CA37912 and by the Bruce McMilan, Jr., Foundation.

*(Received December 1, 1995)*

*(Accepted February 14, 1996)*

### REFERENCES

Anderson G., Anderson K.L., Conroy L.A., Hallam T.J., Moore N.C., Owen J.J.T., and Jenkinson E.J. (1995). Intracellular signaling events during positive and negative selection of CD4$^+$CD8$^+$ thymocytes *in vivo*. J. Immunol. **154**: 3636–3643.

Ashton-Rickardt P.G., Bandeira A., Delaney J.R., Van Kaer L., Pircher H.-P., Zinkernagel R.M., and Tonegawa S. (1994). Evidence for a differential avidity model of T cell selection in the thymus. Cell **76**: 651–663.

Cairns J.S., Mainwaring M.S., Cacchione R.N., Walker J.A., and McCarthy S.A. (1993). Regulation of apoptosis in thymocytes. Thymus **21**: 177–193.

Carlow D.A., vanOers N.S.C., Teh S.-J., and Teh H.-S. (1992). Deletion of antigen-specific immature thymocytes by dendritic cells requires LFA-1/ICAM interactions. J. Immunol. **148**: 1595–1603.

Cohen P. (1989). The structure and regulation of protein phosphatases. Annu. Rev. Biochem. **58**: 453–508.

Curnow S.J., and Schmitt-Verhulst A.-M. (1994). The balance between deletion and activation of CD4$^+$8$^+$ thymocytes is controlled by T cell receptor-antigen interactions and is affected by cyclosporin A. Eur. J. Immunol. **24**: 2401–2409.

Dhein J., Walczak H., Baumier C., Debatin K.-M., and Krammer P.H. (1995). Autocrine T-cell suicide mediated by APO-1 (Fas/CD95). Nature **373**: 438–441.

Dudley E.C., Petrie H.T., Shah L.M., Owen J.J., and Hayday A.C. (1994). T cell receptor β chain gene rearrangement and selection during thymocyte development in adult mice. Immunity **1**: 83–93.

Fabien N.H., Auger C., Moreira A., and Monier J.C. (1992). Effects of cyclosporin A on mouse thymus: Immunochemical and ultrastructural studies. Thymus **20**: 153–162.

Fowlkes B.J., Schwartz R., and Pardoll D.M. (1988). Deletion of self-reactive thymocytes occurs at a CD4$^+$CD8$^+$ precursor stage. Nature **334**: 629–623.

Gao E.-K., Lo D., Cheney R., Kanagawa O., and Sprent J. (1988). Abnormal differentiation of thymocytes in mice treated with cyclosporin. A. Nature **336**: 176–179.

Gorczyca W., Gong J., and Darzynkiewicz Z. (1993). Detection of DNA strand breaks in individual apoptotic cells by the in situ terminal deoxynucelotidyl transferase and nick translation assay. Cancer Res. **5**: 1945–1951.

Hogquist K.A., Jameson S.C., Heath W.R., Howard J.L., Bevan M.J., and Carbone F.R. (1994). T cell receptor antagonist peptides induce positive selection. Cell **76**: 17–27.

Iwabuchi K., Nakayama K.-I., McCoy R.L., Wang F., Nishimura T., Habu S., Murphy K.M., and Loy D.Y. (1992). Cellular and peptide requirements for *in vitro* clonal deletion of immature thymocytes. Proc. Natl Acad. Sci. USA **89**: 9000–9004.

Jenkins M.K., Schwartz R.H., and Pardoll D.M. (1988). Effects of cyclosporine A on T cell development and clonal delection. Science **241**: 1655–1658.

Kearse K.P., Roberts J.L., Munitz T.I., Weist D.L., Nakayama T., and Singer A. (1994). Developmental regulation of αβ T-cell antigen receptor expression results from differential stability of nascent TCRα proteins within the endoplasmic reticulum of immature and mature T cells. EMBO J. **13**: 4504–4514.

Kearse K.P., Takahama Y., Punt J.A., Sharow S.O., and Singer A. (1995). Early molecular events induced by T cell receptor (TCR) signaling in immature CD4$^+$8$^+$ thymocytes: Increased synthesis of TCR-α protein is an early response to TCR signaling that compensates for TCR-α instability, improves TCR assembly, and parallels other indicators of positive selection. J. Exp. Med. **181**: 193–202.

Kishimoto H., Surh C.D., and Sprent J. (1995). Upregulation of surface markers on dying thymocytes. J. Exp. Med. **181**: 649–655.

Kisielow P., Bluthmann H., Staerz U.D., Steinmetz M., and von Boehmer H. (1988). Tolerance in T-cell-receptor transgenic mice involves deletion of nonmature CD4$^+$CD8$^+$ precursor stage. Nature **333**: 742–746.

Kizaki H., Tadakuma T., Odaka C., Muramatsu J., and Ishimura Y. (1989). Activation of a suicide process of thymocytes through DNA fragmentation by calcium ionophores and phorbol esters. J. Immunol. **143**: 1790–1794.

McCaffrey P.G., Perrino B.A., Soderling T.R., and Rao A. (1993). NF-$AT_p$, a T lymphocyte DNA-binding protein that is a target for calcineurin and immunosuppressive drugs. J. Biol. Chem. **268**: 3747–3752.

McCarthy S.A., Cacchine R.N., Mainwaring M.S., and Cairns J.S. (1992). The effects of immunosuppressive drugs on the regulation of activation-induced apoptotic cell death in thymocytes. Transplantation **54**: 543–547.

McConkey D.J., Fosdick L., D'Adamio L., Jondal M., and Orrenius S. (1994). Co-receptor (CD4/CD8) engagement enhances CD3-induced apoptosis in thymocytes. J. Immunol. **153**: 2436–2443.

McConkey D.J., Hartzell P., Amador-Perez J.F., Orrenius S., and Jondal M. (1989a). Calcium dependent killing of immature thymocytes by stimulation via the CD3/T cell receptor complex. J. Immunol. **143**: 1801–1806.

McConkey D.J., Nicotera P., Hartzell P., Bellomo G., Wyllie A.H., and Orrenius S. (1989b). Glucocorticoids activate a suicide process in thymocytes through an elevation of cytosolic $Ca^{2+}$ concentration. Arch. Biochem. Biophys. **269**: 365–370.

Migita K., Eguchi K., Kawabe Y., Mizokami A., Tsukada T., and Nagataki S. (1994). Prevention of anti-CD3 monoclonal antibody-induced thymic apoptosis by protein tyrosine kinase inhibitors. J. Immunol **153**: 3457–3465.

Mombaerts P., Clarke A.R., Rudnicki M.A., Iacomini J., Itohara S., Lafaille J.J., Wang L., Ichikawa Y., Jaenisch R., Hooper M.L., and Tonegawa S. (1992). Mutations in T-cell antigen receptor genes α and β block thymocyte development at different stages. Nature **360**: 225–231.

Nikolic-Zugic J., and Bean M.J. (1988). Thymocytes expressing CD8 differentiate into CD4$^+$ cells following intrathymic injection. Proc. Natl Acad. Sci. USA **85**: 8633–8637.

Ogasawara J., Suda T., and Nagata S. (1995). Selective apoptosis of CD4$^+$CD8$^+$ thymocytes by the anti-Fas antibody. J. Exp. Med. **181**: 485–491.

Page D.M., Kane L.P., Allison J.P., and Hedrick S.M. (1993). Two signals are required for negative selection of CD4$^+$8$^+$ thymocytes. J. Immunol. **151**: 1868–1880.

Philpott K.L., Viney J.L., Kay G., Rastan S., Gardiner E.M., Chae S., Hayday A.C., and Owen M.J. (1992). Lymphoid development in mice congenitally lacking T cell receptor αβ-expressing cells. Science **256**: 1448–1452.

Punt J.A., Osborne B.A., Takahama Y., Sharrow S.O., and Singer A. (1994). Negative selection of CD4$^+$8$^+$ thymocytes by T cell receptor-induced apoptosis requires a costimulatory signal that can be provided by CD28. J. Exp. Med. **170**: 709–713.

Robey E., and Fowlkes B.J (1994). Selective events in T cell development. Annu. Rev. Immunol. **12**: 675–705.

Schreiber S.L., and Crabtree G.R. (1992). The mechanism of action of cyclosporin A and FK506. Immunol. Today **13**: 136–142.

Sentman C.L., Shutter J.R., Hockenberry D., Kanagawa O., and Korsmeyer S.J. (1991). *bcl*-2 inhibits multiple forms of apoptosis but not negative selection in thymocytes. Cell **67**: 879–888.

Shi Y., Bissonnette R.P., Parfrey N., Szalay M., Kubo R., and Green D.R. (1991). In vivo administration of monoclonal antibodies to the CD3 T cell receptor complex induces cell death (apoptosis) in immature thymocytes. J. Immunol. **146**: 3340–3346.

Shi Y., Sahai B.M., and Green D.R. (1989). Cyclosporin A inhibits activation-induced cell death in T-cell hybridomas and thymocytes. Nature **339**: 625–626.

Siegel R.M. Katsumata M., Miyashita T., Louie D.C., Greene M.I., and Reed J.C. (1992). Inhibition of thymocyte apoptosis and negative antigenic selection in *bcl*-2 transgenic mice. Proc. Natl Acad. Sci. USA **89**: 7003–7007.

Smith C.A., Williams G.T., Kingston R., Jenkinson E.J., and Owen J.J.T. (1989). Antibodies to CD3/TCR complex induced death by apoptosis in immature T cells in thymic cultures. Nature **337**: 181–184.

Strasser A., Harris A.W., and Cory S. (1991). *bcl*-2 transgene inhibits T cell death and perturbs thymic self-censorship. Cell **67**: 889–899.

Surh C., and Sprent J. (1994). T-cell apoptosis detected *in situ* during positive and negative selection in the thymus. Nature **372**: 100–103.

Swat W., Dessing M., von Boehmer H., and Kisielow P. (1993). CD69 expression during selection and maturation of CD4+8+ thymocytes. Eur. J. Immunol. **23**: 739–746.

Swat W., Ignatowicz L., von Boehmer H., and Kisielow P. (1991). Clonal deletion of immature CD4+8+ thymocytes in suspension culture by extrathymic antigen presenting – cells. Nature **351**: 150–153.

Takahashi T., Tanaka M., Brannan C.I., Copeland N.G., Jenkins N.A., Suda T., and Nagata S. (1994). Generalized lymphoproliferative disease in mice caused by a point mutation in the Fas ligand. Cell **76**: 969–976.

Ucker D.S., Ashwell J.D., and Nickas G. (1989). Activation driven T cell death. I. Requirements for de novo transcription and translation and association with genome fragmentation. J. Immunol. **143**: 3461–3469.

Urdahl K.B., Pardoll D.M., and Jenkins M.K. (1994). Cyclosporin A inhibits positive selection and delays negative selection in αβ TCR transgenic mice. J. Immunol. **152**: 2853–2859.

Vasquez N.J., Kaye J., and Hedrick S.M. (1992). *In vivo* and *in vitro* clonal deletion of double-positive thymocytes. J. Exp. Med. **175**: 1307–1316.

Vasquez N.J., Kane L.P., and Hedrick S.M. (1994). Intracellular signals that mediate thymic negative selection. Immunity **1**: 45–56.

Venkataraman L., Burakoff S.J., and Sen R. (1995). FK506 inhibits antigen receptor-mediated induction of c-*rel* in B and T lymphoid cells. J. Exp. Med. **181**: 1091–1099.

von Boehmer H. (1994). Positive selection of lymphocytes. Cell **76**: 219–228.

Wang C.-R., Hashimoto K., Kubo S., Yokochi T., Kubo M., Suzuki M., Suzuki K., Tada T., and Nakayama T. (1995). T cell receptor-mediated signaling events in CD4+CD8+ thymocytes undergoing thymic selection: Requirement for calcineurin activation for thymic positive selection but not negative selection. J. Exp. Med. **181**: 927–941.

Watanabe-Fukkunaga R., Brannon C.I., Copeland N.G., Jenkins N.A., and Nagata S. (1992). Lymphoproliferation disorder in mice explained by defects in Fas antigen that mediates apoptosis. Nature **356**: 314–317.

Zacharchuk C.M., Mercep M., Chakraborti P.K., Simons S.S., and Ashwell J.D. (1990). Programmed T lymphocyte death: Cell activation- and steroid-induced pathways are mutually antagonistic. J. Immunol. **145**: 4037–4045.

